# Reliability of the biceps brachii M-wave

**DOI:** 10.1186/1743-0003-2-33

**Published:** 2005-12-06

**Authors:** Kristina M Calder, Lesley-Ann Hall, Steve M Lester, J Greig Inglis, David A Gabriel

**Affiliations:** 1 Electromyographic Kinesiology Laboratory, Faculty of Applied Helath Science, Brock University, 500 Glenridge Avenue, St.Catharines, Ontario, L2S 3A1 Canader

**Keywords:** Compound muscle action potential, intraclass correlation coefficient, electromyographic activity

## Abstract

**Background:**

The peak-to-peak (P-P) amplitude of the maximum M-wave and the area of the negative phase of the curve are important measures that serve as methodological controls in H-reflex studies, motor unit number estimation (MUNE) procedures, and normalization factors for voluntary electromyographic (EMG) activity. These methodologies assume, with little evidence, that M-wave variability is minimal. This study therefore examined the intraclass reliability of these measures for the biceps brachii.

**Methods:**

Twenty-two healthy adults (4 males and 18 females) participated in 5 separate days of electrical stimulation of the musculocutaneous nerve supplying the biceps brachii muscle. A total of 10 stimulations were recorded on each of the 5 test sessions: a total of fifty trials were used for analysis. A two-factor repeated measures analysis of variance (ANOVA) evaluated the stability of the group means across test sessions. The consistency of scores within individuals was determined by calculating the intraclass correlation coefficient (ICC). The variance ratio (VR) was then used to assess the reproducibility of the shape of the maximum M-wave within individual subjects.

**Results:**

The P-P amplitude means ranged from 12.62 ± 4.33 mV to 13.45 ± 4.07 mV across test sessions. The group means were highly stable. ICC analysis also revealed that the scores were very consistent (ICC = 0.98). The group means for the area of the negative phase of the maximum M-wave were also stable (117 to 126 mV·ms). The ICC analysis also indicated a high degree of consistency (ICC = 0.96). The VR for the sample was 0.244 ± 0.169, which suggests that the biceps brachii maximum M-wave shape was in general very reproducible for each subject.

**Conclusion:**

The results support the use of P-P amplitude of the maximum M-wave as a methodological control in H-reflex studies, and as a normalization factor for voluntary EMG. The area of the negative phase of the maximum M-wave is both stable and consistent, and the shape of the entire waveform is highly reproducible and may be used for MUNE procedures.

## Background

The massed action potential (M-wave) is known as the compound muscle action potential (CMAP); it is an important investigative tool in several different areas of neurophysiological research. Evoking the maximum M-wave (M_max_) by supramaximal stimulation is the electrical equivalent of the recruitment of all motor units within the motor neuron pool [1]. The M_max _is a methodological control to ensure that the effective stimulus intensity to peripheral nerves is consistent across recording sessions [2]. Using a stimulus intensity that produces M-wave responses corresponding to a consistent percentage of M_max _ensures that the same numbers of motor axons are recruited in each trial [3].

The area of the negative phase of the maximum M-wave is a critical part of motor unit number estimation for tracking the progression of neuromuscular disorders [4]. The P-P amplitude of the maximum M-wave is used in Hoffman reflex (H-reflex) studies to accurately conclude that variations in the H-reflex arise from a neural origin, and are not caused by changes in the muscle, recording conditions, or problems with instrumentation [3]. This is accomplished by calculating the ratio between the maximum P-P amplitude of the H-reflex and the M-wave (H_max_/M_max_), which is considered an index of excitability of the H-reflex arc [1,3,5]. Similarly, the M-wave is also used as a normalization factor to correct for day-to-day fluctuations in voluntary electromyographic (EMG) activity due to slight differences in electrode placement, muscle temperature, and other such considerations [6,7].

The methodologies described above are based on the assumption that there is little variability in the M-wave. Given the importance of the M-wave as a clinical and investigative tool, there are surprisingly few studies that have documented the variability of this waveform. Studies thus far have only examined simple test-retest reliability [4,8-10], and only one of these studies has included both the P-P amplitude and the area of the negative phase of the maximum M-wave [3]. Two studies have performed a more comprehensive analysis of M-wave reliability using the intraclass correlation coefficient (ICC), but neither the P-P amplitude nor area of the negative phase of the maximum M-wave were specifically investigated [11,12]. The purpose of this study was therefore to examine maximum M-wave reliability using both the P-P amplitude and area of the negative of the curve for responses obtained from the biceps brachii muscle over a series of five test sessions.

## Materials and methods

### Subjects

A total of 22 healthy subjects participated in this study. Table 1 provides data for the demographic characteristics of the subjects. An informed consent form was read and signed prior to participation, in accordance with Brock University's human ethics.

**Table 1:** Physical characteristics of the subjects (N = 22) including gender, age, height, weight and body mass index (BMI).

Subject no.	Gender	Age (years)	Height (m)	Weight (kg)	BMI (kg/m^2^)
1	F	24	1.70	68.04	23.49
2	F	28	1.70	62.60	21.61
3	F	22	1.56	66.68	27.40
4	F	25	1.60	47.00	18.36
5	F	27	1.60	72.12	28.17
6	F	21	1.68	68.04	24.21
7	F	31	1.63	49.90	18.88
8	F	20	1.58	56.70	22.86
9	F	22	1.65	53.52	19.64
10	F	22	1.68	52.16	18.56
11	F	22	1.73	56.70	19.01
12	F	18	1.75	66.68	21.71
13	F	19	1.70	58.97	20.36
14	F	22	1.68	72.58	25.71
15	M	18	1.82	82.56	24.92
16	F	27	1.78	76.20	24.05
17	F	20	1.65	56.25	20.66
18	F	20	1.61	46.27	17.85
19	M	29	1.83	97.07	28.99
20	F	22	1.71	58.97	20.17
21	M	26	1.96	104.33	27.16
22	M	20	1.72	67.00	22.65

Mean ± SD		22.87 ± 3.61	1.69 ± 0.09	65.05 ± 14.65	22.50 ± 3.35

### Measurement schedule and procedures

There were 5 days of testing with at least 24 hours between each session. All testing was done on the right arm while subjects lay prone on a gurney with their shoulder abducted to 90° at their side, palm facing up with the elbow slightly flexed. Prior to electrode placement, the skin on the right upper arm was lightly abraded with and cleaned with rubbing alcohol to reduce signal impedance at the skin surface. The motor point was then determined for electrode placement. The motor point is defined as the region of the muscle where the lowest possible stimulus will produce a minimal muscle twitch. The motor point of the biceps brachii (BB) was located approximately midway between the glenohumeral joint and the cubital crease.

The cathode portion of the stimulating probe was placed in the estimated motor point region. With the train rate on the stimulator set at 10 pps, and the stimulus duration set at 1 msec [13], the cathode was moved around the muscle belly to find the motor point. Prior to placing the recording (G2) and reference (Gl) electrodes, skin impedance was measured (Grass EZM Electrode Impedance Meter, Astro-Med Inc., Warwick, RI) and maintained below 10 kΩ. The G2 electrode was placed directly above the motor point of the BB muscle. The Gl electrode was placed on the biceps tendon. Both Gl and G2 were standard size (20 mm diameter) Ag/AgCl electrodes (Grass F-E9-40-5, Astro-Med Inc., Warwick, RI). A self-adhesive ground electrode was placed on the upper portion of the biceps muscle, between G2 and the point of stimulation on the musculocutaneous nerve. The EMG system (Grass, P511, Astro-Med Inc., W Warwick, RI) amplified the evoked potentials (1000×) before they were band-passed filtered (3–1000 Hz).

**Figure 1:**
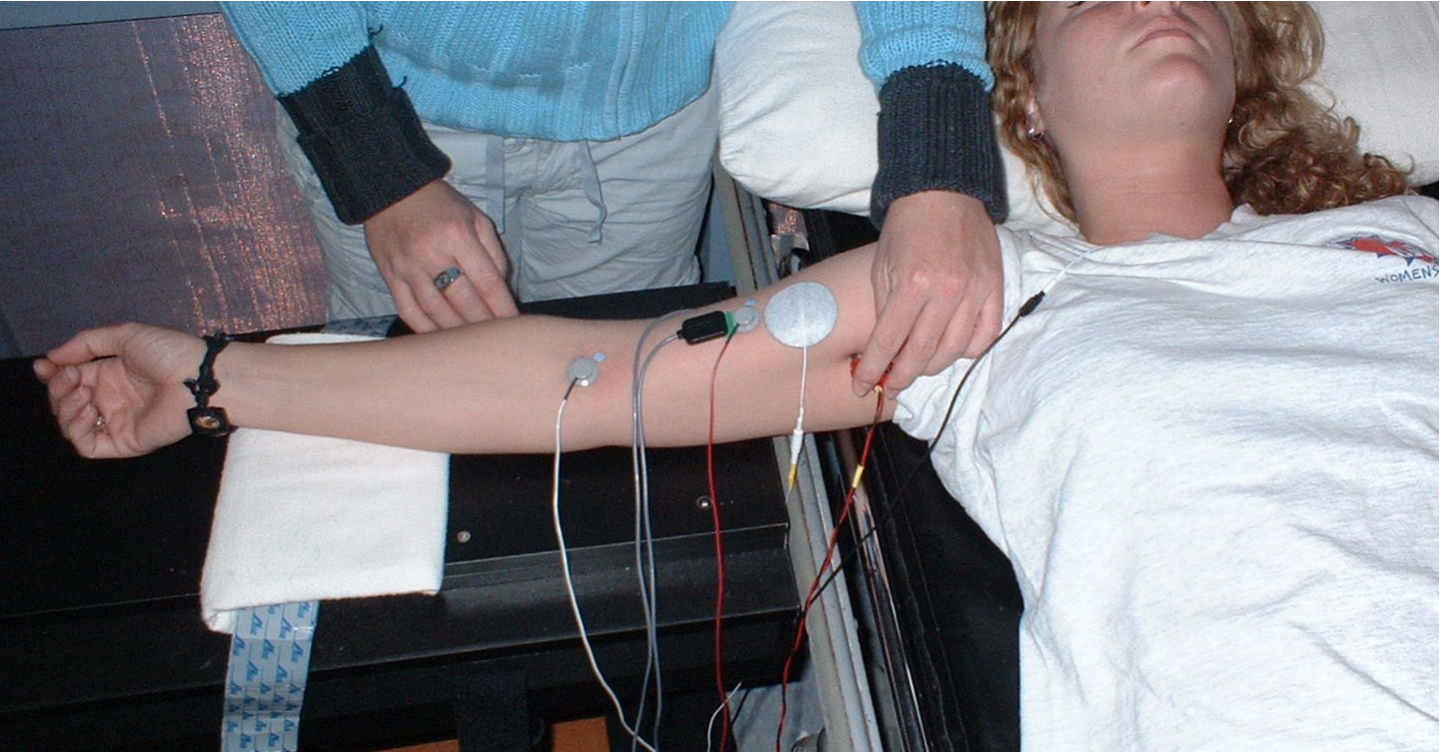
Experimental set-up for stimulation at the musculocutaneous nerve to record maximum M-wave responses from the biceps brachii muscle.

The M-wave was evoked with a cathode placed in the axillary fold over the musculocutaneous nerve (see Figure 1). The cathode and anode electrodes were connected in series with an isolation unit (Grass Telefactor SUI8, Astro-Med, Inc., West Warwick, RI) and a stimulator (Grass Telefactor S88, Astro-Med Inc., West Warwick, RI) that delivered a square-wave pulse, 1 msec in duration [13].

To ensure that electrical stimulation was accurately over the musculocutaneous nerve, and that the BB was the only muscle being activated, bipolar surface electrodes (DE-2.1, Delsys Inc., Boston, MA) were positioned over the biceps and triceps brachii. One electrode was placed on the lower third of the biceps belly, below the motor point towards the distal tendon. The other was placed between the distal tendon and the top of the belly of the triceps lateral head to monitor activity in the antagonist muscles. A self-adhesive ground electrode was also secured over the collarbone. These signals were amplified with a fixed gain of 10 at the skin surface. The EMG system (Bagnoli 4, Delsys Inc., Boston, MA) further amplified the signals (100×) before they were band-passed filtered (20–450 Hz). Electrode placement remained consistent by tracing all sites with indelible ink, and asking participants to preserve these markings for the duration of the study.

All signals were sent to a 16-bit A/D converter (BNC-2110, National Instruments), and sampled at 2048 Hz using a Computer-Based Oscillograph and Data Acquisition System (DASYLab, DASYTEC National Instruments, Amherst, NH). This recorded data was stored for further analysis on a Pentium III PC (Seanix Technology Inc., Blaine, WA).

### Stimulation protocol

Subjects were instructed to close their eyes and lay still throughout the session. The BB M-wave was obtained by stimulating the musculocutaneous nerve as depicted in Figure 1. Stimulations started below the response threshold, and increased in 4 V increments until M_max _was achieved. Stimulus intensity was then increased slightly beyond this point to confirm no further enlargement in the peak-to-peak amplitude, and subsequently returned to the lower intensity where the M response remained stable [14,15]. Ten stimuli were delivered, separated by a 15-second rest after each pulse. The M-waves were recorded for each trial and saved for later analysis. This protocol was followed for each of the 5 days of testing.

### Data reduction

Data reduction was conducted for all ten trials for each of the five test sessions. The P-P amplitude of the biceps brachii M-wave was calculated as the difference between the maximum and minimum of the signal. Area of the negative phase of the biceps brachii M-wave was calculated using trapezoidal integration:



where n is the number of data points, y_i _is the data value at time t, and Δt is the sampling interval. The start of the negative phase of the biceps brachii M-wave was defined as the first point to cross the zero baseline after the stimulus artefact (t_1_, y_1_). The end of the negative phase of the biceps brachii M-wave was the last point before the second baseline crossing (t_n_, y_n_). Since trapezoidal integration is sensitive to interval width (Δt), the entire waveform was interpolated to a sampling rate of 10 kHz prior to calculating area under the curve [11]. All data reduction was completed using MATLAB software (The Mathworks Inc., Natick, MA).

### Analysis

All statistical procedures were performed in SYSTAT (SPSS Inc., Chicago, IL). A significance level of *P <*0.05 was adopted for this study.

### Intraclass correlation analysis of variance

Reliability analysis with the intraclass correlation coefficient (ICC) requires two different analysis of variance (ANOVA) models [15-18]. One is to establish the "consistency" of the measures. This is a fully nested model wherein trials are nested within days, which are in turn nested within subjects. When subjects are able to reproduce their own score, the scores are tightly group around the subject's own mean. In this way, the scores of one subject are very different from the scores of another, and the between subjects means squares (MS) error is high. This is also reflected as a high true score variance (σ^2^_True_), as outlined below. Measures that are highly reliable have a true score variance that accounts for the greatest percentage of the total variance. The second ANOVA model is used to examine the "stability" of the means across test sessions. This ANOVA model has two factors (days × subjects). The repeated measurements (trials) on each subject in each day constituted a "within-cells" replication of measures [15-18]. A measure must therefore exhibit both consistency and stability to be considered reliable. The ICC was calculated in the following way:









The mean square (MS) errors for subjects, days and trials were extracted from the fully nested ANOVA model to calculate the ICC in equation 2. In equations 3–5, a' is number of days, n' is number of trials, σ^2^_e2 _is error variance due to days, σ^2^_e1 _is error variance due to trials, and σ^2^_true _is the true score variance. The total variance σ^2^_Total _was then calculated as the sum of the variances (σ^2^_true _+ σ^2^_e1_+ σ^2^_e2_). The portion of variances attributable to day-to-day (σ^2^_e2_/σ^2^_Total_), trial-to-trial (σ^2^_e1_/σ^2^_Total_), and between subjects (σ^2^_True_/σ^2^_Total_) error were computed to identify the amount of variability at each level of measurement [11,12].

### Variance ratio

The ICC is a ratio of variance due to differences between subjects (signal) to the total variability in the data (signal and noise). Thus, the ICC is a relative measure of the ability to differentiate between individuals [19]. Since clinical measures and normalization techniques are relative to the individual, there is need for an additional measure that assesses the reliability of the biceps brachii M-wave within the individual. The variance ratio (VR) assesses the "reproducibility" of waveform shape for an individual subject [20,21]. The more similar in shape the waveforms are, the variance ratio tends towards 0. The more dissimilar in shape the waveforms are, the variance ratio tends towards 1.

To calculate the VR, the biceps brachii M-wave for each subject had to be normalized in the time-domain to the same number of data points. Only the negative and positive phases of the biceps brachii M-wave were analyzed. The start of the negative phase of the biceps brachii M-wave was defined as the first point to cross the zero baseline after the stimulus artefact (t_1_, y_1_). The end of the positive phase biceps brachii M-wave was the last point before the third baseline crossing (t_n_, y_n_). The waveform between these two points was then interpolated up to 1000 data points (T = 1000). This was done for all 50 waveforms (N = 50) within a subject. The formula for calculating the VR was:



where y_t,n _was the data point for the amplitude of the biceps brachii M-wave at the time t. To calculate , the biceps brachii M-wave was averaged across the 50 trials, which was still a 1000 point waveform. The grand mean  was then a single number that represents the mean of all data points across the 50 trials.

## Results

The means, standard deviations, and *F*-ratios used to evaluate the stability of the P-P amplitude of the maximum M-wave are presented in Table 2. The between-subjects main effect was significant, as was the slight increase (4.3%) in P-P amplitude across test sessions. The day × subjects interaction term was also significant, indicating that not all subjects exhibited the same magnitude of increase in P-P amplitude across test sessions.

**Table 2:** The means (M) and standard deviations (SD) and analysis of variance (ANOVA) F-ratios for peak-to-peak (PP) amplitude and area of the negative phase of the biceps brachii maximum M-wave the for the ten trials across five days for all subjects (N = 22).

Days		P-P Amplitude (mV) (M ± SD)	Area (mV·ms) (M ± SD)
1		12.90 ± 4.64	125.8 ± 40.1
2		12.63 ± 4.25	122.6 ± 40.1
3		12.66 ± 4.22	122.7 ± 39.5
4		12.62 ± 4.33	117.1 ± 37.4
5		13.45 ± 4.07	126.0 ± 35.2
Minimum – Maximum		5.07 – 23.64 mV	33.7 – 208.9 mV·ms
			
Source of Variation	*df*	*F*	*F*
Subjects	21	2120.52^†^	1191.54^†^
Day	4	64.68^†^	39.57^†^
Day × Subjects	84	31.74^†^	29.732^†^
Within Cell	990		

^† ^Significant at the 0.01 level

**Figure 2:**
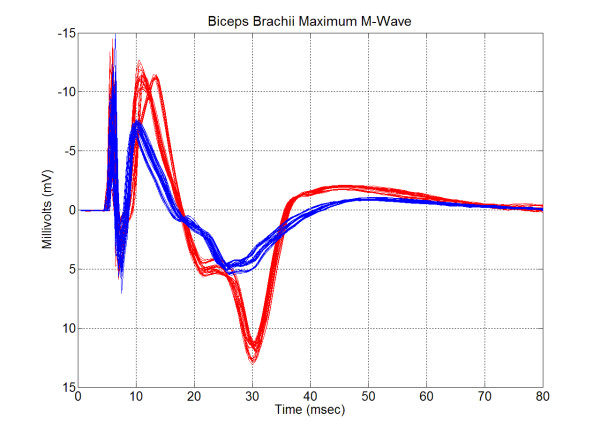
Sample M-wave recordings from the bicep brachii muscle for two subjects. Shown are the ten trials for each of the five test sessions, for a total of fifty waveforms for each subject.

The consistency of the P-P amplitude for the maximum M-wave was evaluated using the intraclass correlation analysis of variance. Despite the slight lack of stability in the group means, individuals exhibited remarkable consistency across test sessions. When individual subjects produce consistent scores, the differences between subjects become evident, yielding a large between-subjects main effect. The resulting between-subjects variability (σ^2^_True_) accounted for 91% of the total variance. This is a prerequisite for high reliability. Figure 2 illustrates how responses between subjects can be very different. At the same time, subjects can exhibit remarkable consistency over trials and across days. The percentage of the variance attributed to trial-to-trial variability (σ^2^_e1_) was 1%, which was much lower than the 8% day-to-day variability (σ^2^_e2_). The ICC for the P-P amplitude was 0.98.

The between-subjects main effect was significant for the area of the negative phase of the biceps brachii M-wave (see Table 2). There was a significant main effect for days as the area measure was also 4 to 7% lower on Day 4 than on any other day. The day × subjects interaction term was significant, indicating that not all subjects exhibited same pattern of change in the area measure across test sessions. However, this slight lack of stability in group means was compensated for by a high degree of consistency within subjects. The between subjects variability (σ^2^_True_) accounted for 80% of the total variance, which is necessary for a high ICC. The trial-to-trial variability (σ^2^_e1_) was 5% while the day-to-day variability (σ^2^_e2_) was three-fold greater (15%). The resulting ICC was 0.96.

**Figure 3:**
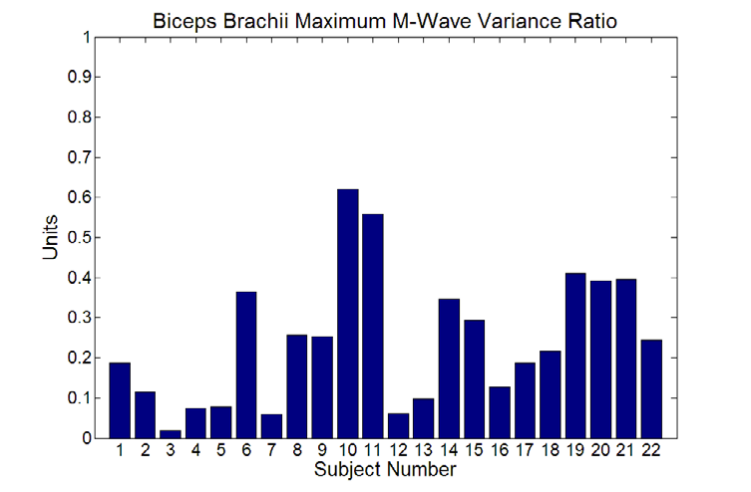
The variance ratios (VR) for all 22 subjects.

**Figure 4:**
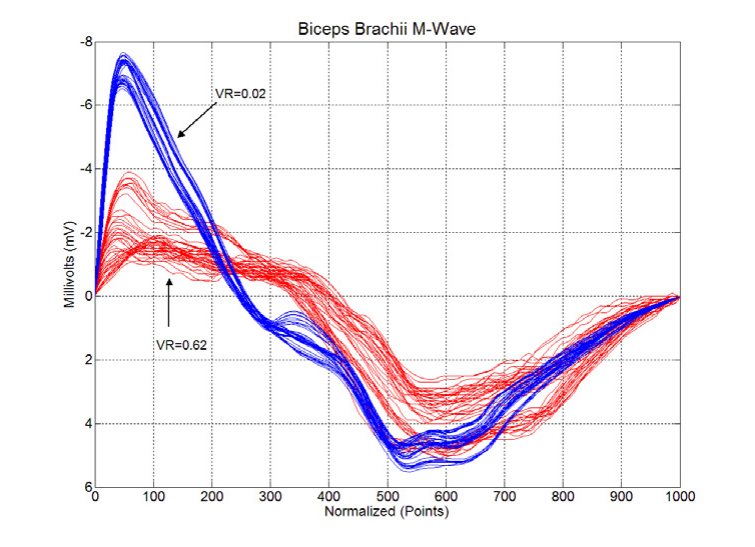
Time-domain normalized maximum M-wave recordings from the bicep brachii muscle for two subjects. Shown are the ten trials for each of the five test sessions, for a total of fifty waveforms for each subject. The two sets of waveforms illustrate the extreme range of variance ratios observed in this study.

The VR for the sample was 0.244 ± 0.169, indicating that the biceps brachii maximum M-wave shape was in general very reproducible for each subject. There was of course a range of VRs. The biceps brachii maximum M-wave shape was less reproducible for some subjects than others, but they were few (see Figure 3). Figure 4 presents the waveforms associated with the two extremes observed in this study.

## Discussion

The reliability the P-P amplitude and the area of the negative phase of the maximum M-wave was assessed for fifty trials distributed equally across five test sessions. The P-P amplitude of the maximum M-wave exhibited excellent reliability (ICC = 0.98) and so did the area of the negative phase of the maximum M-wave (ICC = 0.96). These high ICC values are consistent with previous work from our laboratory, on other muscle groups [14,15]. In the following paragraphs we will discuss the theoretical implications and practical application of our results.

The P-P amplitude of the maximum M-wave ranged from 12.62 ± 4.33 mV to 13.45 ± 4.07 mV across test days. Taylor et al. [22] reported a mean of 13.4 ± 4.2 mV while Allman and Rice [23] observed a mean of 15.3 ± 5.6 mV. Thus, our results are well within the range of values found in the literature. The area of the negative phase of the maximum M-wave is used for MUNE, but its value in absolute units is seldom reported. To the best of our knowledge, no comparative data exist for the biceps brachii. In this respect, the current work contributes normative data to the existing literature. Rutkove [24] reported a mean of 44.2 mV·ms for the abductor pollicis (thenar muscle) while Boe et al. [4] observed a nearly identical value (44.5 mV·ms). The later research group [4] also found a mean of 29.2 mV·ms for the first dorsal interosseus/adductor pollicis muscle. The biceps brachii means observed here ranged from 117.1 to 126.0 mV·ms. Given the large P-P amplitude values and longer durations compared to smaller muscles, the area values are quite reasonable.

The ICC analysis of variance technique resulted in a reliability coefficient for the P-P amplitude of the maximum M-wave that was excellent. A high reliability is obtained when the between-subjects variance is substantially larger than the variance in scores within subjects, and the variance of scores due to error is minimized. Individual subjects in the present study exhibited highly consistent P-P amplitude scores, so that the variation in scores between subjects could be clearly observed. Thus, the high ICC value indicates that the P-P amplitude of the maximum M-wave was a reliable estimation of complete activation of the associated the motoneuron pool. These findings provide evidence that support the use of the P-P amplitude of the maximum M-wave as a methodological standard against which other muscular responses, such as the H-reflex or voluntary EMG can be assessed.

The area of the negative phase of the maximum M-wave also exhibited excellent reliability for the same reasons as P-P amplitude. The maximum M-wave represents complete activation of the muscle associated with the stimulated peripheral nerve [25]. In the absence of a neuromuscular disorder, it should remain unchanged over time as the number of α-motoneurons remains constant [3]. It is unreasonable to expect that any physiological measure would have perfect reliability, but an ICC of 0.96 does indicate that the area of the negative phase of the maximum M-wave can be a stable and consistent measure of the number of α-motoneurons. The results presented here therefore support its use in MUNE.

To date, reliability studies on MUNE have utilized quite limited statistical techniques. The Pearson correlation coefficient and the *t*-test were combined to evaluate MUNE values obtained on only two separate occasions [4,9]. Both methods combined are limited in that they are still insensitive to the problem of consistency. One study did use the CV to evaluate the consistency of individual subjects, but the stability of the group means was not considered [26]. The current study presents a comprehensive treatment of the reliability of the area of the negative phase of the maximum M-wave. This is important as MUNE is performed over multiple test sessions to monitor the progression of neuromuscular disorders.

Other investigators have reported that trial-to-trial variability accounted for the lowest percentage of the total variance [11,12]. The peripheral nerve was recruited by a hand-held stimulator as would occur during clinical testing [27]. The low trial-to-trial variability indicates that this was not an issue in the present investigation. As might be expected, multiple test sessions introduce additional sources of error. There could be slight differences in the position of the stimulating and recording electrodes, changes in electrode-skin input impedance and/or muscle temperature [11,12,27]. Limb position is also critical as it alters position of the nerve relative to the skin surface, making it harder or easier to evoke the potential [27]. Changes in muscle geometry relative to the skin surface associated different limb positions can alter the shape of the evoked potential [12,27]. The low day-to-day variance observed in this study suggests that careful methodological controls can minimize these potential sources of error.

The resulting ICC values are higher in the current work versus previous publications [11,12]. The reason may be due to fundamentally different methodologies.  In addition to well-controlled electrode placement and methodology, there was a strict adherence to a well-documented anatomic reference position and stimulation site for the peripheral nerve.  Stimulation of the peripheral nerve and recording the response at the measured motor point are key to obtaining crisp, reliable M-waves.  Previous ICC studies [11,12] use a non-clinical protocol.  The two papers [11,12] use electrical stimulation of the motor point and recording the M-wave between the motor point and distal tendon.  The recorded M-wave is more susceptible to distortions associated with temporal dispersion and a contracting muscle; it could not be used for MUNE.

Figure 2 was used to illustrate the relative nature of the ICC. Individual responses can have a certain degree of variability, but, if differences between subjects can be detected, the ICC will be high. Thus, the VR was included in this study to assess reproducibility of M-wave shape for individual subjects. There is no generally accepted delineation of excellent or even acceptable ranges of VR as exists with the ICC. Jacoboson et al. [21] reviewed the existing literature and set an upper limit of 0.40 as the criteria below which the same muscle group on the right and left legs would exhibit symmetrical profiles for linear envelop detected EMG during gait. In the current sample, two subjects had a VR much greater than 0.40 while a third was only slightly greater than 0.40. The remainder of the sample had VRs below 0.40. The results for the ICC and VR taken together support the earlier observation of Merletti et al. [11,12] that shape features of M-waves are so reliable that visual identification of subjects is possible based on their M-waves.

## Conclusion

The results support the use of P-P amplitude of the maximum M-wave as a methodological control in H-reflex studies, and as a normalization factor for voluntary EMG. The area of the negative phase of the maximum M-wave is both stable and consistent, and the shape of the entire waveform is highly reproducible and may be used for MUNE procedures. The intraclass correlation analysis of variance is necessary for establishing the reliability (stability and consistency) of EMG waveform measures, but not sufficient for investigating reproducibility of the EMG waveform shape. The variance ratio demonstrated that the shape of the biceps brachii maximum M-wave was very reproducible for all but a few subjects. Such information is important if the M-wave is to be used in tracking the progression of neuromuscular disorders.
